# Genes and speciation: is it time to abandon the biological species concept?

**DOI:** 10.1093/nsr/nwz220

**Published:** 2019-12-31

**Authors:** Xinfeng Wang, Ziwen He, Suhua Shi, Chung-I Wu

**Affiliations:** State Key Laboratory of Biocontrol, Guangdong Key Lab of Plant Resources, School of Life Sciences, Sun Yat-Sen University, Guangzhou 510275, China; State Key Laboratory of Biocontrol, Guangdong Key Lab of Plant Resources, School of Life Sciences, Sun Yat-Sen University, Guangzhou 510275, China; State Key Laboratory of Biocontrol, Guangdong Key Lab of Plant Resources, School of Life Sciences, Sun Yat-Sen University, Guangzhou 510275, China; State Key Laboratory of Biocontrol, Guangdong Key Lab of Plant Resources, School of Life Sciences, Sun Yat-Sen University, Guangzhou 510275, China; Department of Ecology and Evolution, University of Chicago, Illinois 60637, USA

**Keywords:** speciation, species concept, gene flow, geographical isolation, allopatry

## Abstract

The biological species concept (BSC) is the cornerstone of neo-Darwinian thinking. In BSC, species do not exchange genes either during or after speciation. However, as gene flow during speciation is increasingly being reported in a substantial literature, it seems time to reassess the revered, but often doubted, BSC. Contrary to the common perception, BSC should expect substantial gene flow at the onset of speciation, not least because geographical isolation develops gradually. Although BSC does not stipulate how speciation begins, it does require a sustained period of isolation for speciation to complete its course. Evidence against BSC must demonstrate that the observed gene flow does not merely occur at the onset of speciation but continues until its completion. Importantly, recent genomic analyses cannot reject this more realistic version of BSC, although future analyses may still prove it wrong. The ultimate acceptance or rejection of BSC is not merely about a historical debate; rather, it is about the fundamental nature of species – are species (and, hence, divergent adaptations) driven by a relatively small number of genes, or by thousands of them? Many levels of biology, ranging from taxonomy to biodiversity, depend on this resolution.

## INTRODUCTION

At a very basic level, species may be perceived either as discrete adaptive entities or as mutually exclusive gene pools. In his *Origin of Species*, Darwin [1] portrayed species almost exclusively as adaptive entities. He did devote Chapter 8 to hybrid sterility, which, he noted, is ‘not a specially acquired or endowed quality, but is incidental on other acquired differences.’ In the era of modern synthesis (e.g. [2–8]), the concept of species has shifted to the latter quality, emphasizing species mainly by their incompatibilities. The lament by Darwin that hybrid sterility ‘has been much underrated by late writers’ has been amply rectified (e.g. [9]).

Among the large number of species concepts compiled in recent times [10,11], a few indeed emphasize species being the adaptive units. These include the recognition and cohesion concepts [12,13], as well as the literature devoted to the adaptive aspect of species, for example, on augmenting the genetic variation for adaptation via hybridization [14–16]. Nevertheless, the main concept of species has been centered on the mutual exclusivity between species. The biological species concept (BSC), the best example of the latter, has been the gold standard in the modern era [2–4].

In this article, we will review the modern literature that appears to reject BSC, both in concept and in empirical observations. Despite the overwhelming evidence against it, BSC remains the dominant species concept at present. Its dominance can be seen in the most popular textbooks read by the next generation of evolutionary biologists as if BSC had been proven beyond doubt [3,17]. Clearly, the contradictions need to be resolved. We should emphasize that BSC is far more than an unresolved issue for mere historical reasons. It is fundamental to our understanding of the genetic nature of species; hence, many key evolutionary issues depend on its resolution (see Discussion).

## THE BIOLOGICAL SPECIES CONCEPT

In BSC, species are separated by various forms of reproductive isolation (RI) and do not exchange genes [2–8,18–24]. BSC also denotes a process that permits reproductively incompatible populations to emerge. In this process, the absence of gene flow is essential because genetic exchanges are perceived to be capable of reversing the divergence. BSC makes strong assumptions about the genetics of species divergence, postulating that almost the entire genome evolves as a cohesive unit [2,5]. It is essentially a genomic concept of species and speciation [5].

Once the process of speciation is completed, ‘there can be a little genetic leakage between species through hybridization; [hence] the BSC does not require that species be 100 percent reproductively isolated’ (p. 215 [3]). These exchanges are tolerated, for example, in hybrid zones (p. 217 [3] and [25,26]), *only* because such hybridizations have little consequence on the genetic integrity of the species *already formed*. The post-speciation ‘genetic leakage’ does not mean the tolerance of genetic exchanges during speciation under BSC. Genetic exchange during speciation, like cheating in a class, is delineated between zero and non-zero. (Otherwise, what does a little exchange, or a little cheating, mean?)

Here, we will use the term BSC broadly for both the process of speciation and the concept of species. As BSC demands a prolonged period of divergence during which there is no gene flow, geographical isolation appears to be a most effective mechanism. This mode is generally referred to as allopatric speciation.

Finally, there is a conspicuous but odd fact about BSC: under this concept, species status is often indeterminate when the lack of gene flow is a result of geographical distance. That is one reason why BSC is rarely used in the actual practice of delineating species. Instead, species delineation generally relies on phenotypic criteria such as morphology, ecological preferences, etc. Hence, BSC cannot be defended as a practical, albeit imperfect, concept. It is, instead, a perfectly impractical concept.

## THE ALTERNATIVE ‘GENIC VIEW’ OF SPECIATION (VIS-À-VIS BSC)

In an alternative view, species are defined by a set of loci that govern the morphological, reproductive, behavioral and ecological characters. These ‘speciation genes’ may collectively account for no more than a fraction of the genome [5,27–29]. This fraction should be fitness-reducing on introgression [30], whereas the rest of the genome can be freely exchanged without a fitness consequence. In short, the diverging genomes comprise both introgressable and non-introgressable DNA segments. These non-introgressable segments are often referred to as ‘genomic islands,’ which are, in theory, more divergent than the rest of the genome [5,29–34]. This concept has been referred to as the genic view of speciation [5], in contrast with the genomic perspective of BSC. The genic view of speciation differs from most species concepts as it merges the adaptive view and the isolation concept of species presented in the Introduction.

In the last 20 years, genomic islands have been identified in many speciation events [33–49] (see Table 1 for a compilation). The literature suggests that ‘speciation with gene flow’ is rather common in a wide array of taxa [35–49]. If DNA segments between the genomic islands indeed represent exchanges during speciation, it would seem timely to abandon BSC as the defining concept of species and speciation. Therefore, the issue is whether the genomic evidence on species divergence [35–49] is collectively incompatible with BSC.

**Table 1.** Publications on ‘speciation with gene flow’ since 2005.

	Taxa	Species	Main research focus	Inferred stage of speciation	Reference
1	*Anopheles*	*A. gambiae*	Genomic islands of speciation in *Anopheles gambiae*	**Early stage.** Reason: the materials are two sympatric, partially isolated subtaxa known as M form and S form of *A. gambiae*	Turner *et al*., 2005
2	*Mus*	*M. musculus* and *M. m. domesticus*	Genomic islands of differentiation between house mouse subspecies	**Early stage.** Reason: the materials are subspecies, although there seems to be partial RI associated with the X	Harr, 2006
3	*Ficedula*	*F. albicollis* and *F. hypoleuca*	The genomic landscape of species divergence in *Ficedula* flycatchers	**Uncertain.** Reason: although the age of divergence is said to be >1 Myrs, the fixed differences seem low (see their table 2)	Ellegren *et al*., 2012
4	*Heliconius*	*H. melpomene*, *H. cydno* and *H. timareta*	Genome-wide evidence for speciation with gene flow in *Heliconius* butterflies	**Uncertain.** Reason: the case is commented in the main text. The density of speciation genes seems low	Martin *et al*., 2013
5	*Helianthus*	*H. petiolaris*, *H. debilis*, *H. annuus* and *H. argophyllus*	Genomic islands of divergence are not affected by geography of speciation in sunflowers	**Early stage.** Reason: the materials are four recently diverged pairs of sunflower species with low pair *F_ST_*. There are far fewer fixed changes than polymorphic ones (see their table 2)	Renaut *et al*., 2013
6	*Mimulus*	*M. guttatus* and *M. nasutus*	Speciation and introgression between *Mimulus nasutus* and *Mimulus guttatus*	**Uncertain.** Reason: these two sister species are 200 000–500 000 years apart	Brandvain *et al.*, 2014
7	*Oryctolagus*	*O. cuniculus algirus* and *O. c. cuniculus*	The genomic architecture of population divergence between subspecies of the European Rabbit	**Early stage.** Reason: the materials are two subspecies of rabbits in the early stages of divergence	Carneiro *et al*., 2014
8	*Anopheles*	*A. gambiae* species pair (*A. coluzzii* and *A. gambiae sensu stricto*)	Adaptive introgression between *Anopheles* sibling species eliminates a major genomic island but not reproductive isolation	**Early stage.** Reason: the materials are the M form and S form of *A. gambiae*	Clarkson *et al*., 2014
9	*Corvus*	*C. (corone) corone* and *C. (corone) cornix*	The genomic landscape underlying phenotypic integrity in the face of gene flow in crows	**Early stage.** Reason: the two species show only a small number of narrow genomic islands across the whole genome	Poelstra *et al*., 2014
10	*Anopheles*	*An. gambiae* complex	Extensive introgression in a malaria vector species complex revealed by phylogenomics	**Early stage.** Reason: it is evident that the introgressions in these species are earlier-stage events (see their Fig. 1C)	Fontaine *et al*., 2015
11	Multiple taxa	All Darwin's finch species and two tanagers, *Tiaris bicolor* and *Loxigilla noctis*	Evolution of Darwin's finches and their beaks revealed by genome sequencing	**Early stage.** Reason: they find extensive evidence for interspecific gene flow throughout the radiation which represents the early stages of diversification when phenotypic transitions between species	Lamichhaney *et al*., 2015
12	*Astatotilapia*	Two cichlid fish ecomorphs	Genomic islands of speciation separate cichlid ecomorphs in an East African crater lake	**Early stage.** Reason: this study is about the discovery and detailed characterization of early-stage adaptive divergence of two cichlid fish	Malinsky *et al*., 2015
13	*Vermivora*	*V. chrysoptera* and *V. cyanoptera*	Plumage genes and little else distinguish the genomes of hybridizing warblers	**Early stage.** Reason: the two species show extremely low differentiation: only six small genomic regions exhibit strong differences	Toews *et al*., 2016
14	*Xiphophorus*	Three hybrid pops between sister species of *X. birchmanni* and *X. malinche*	Natural selection interacts with recombination to shape the evolution of hybrid genomes	**Early stage.** Reason: they studied three replicate hybrid populations that formed naturally between two sister swordtail fish species	Schumer *et al*., 2018
15	*Heliconius*	Many *Heliconius* butterflies species	Genomic architecture and introgression shape a butterfly radiation	**Early stage.** Reason: introgressions happened during the process of adaptive radiation	Edelman *et al*., 2019

## AN EXPANDED BSC THAT PERMITS GENE FLOW IN A TRANSIENT EPISODE AT THE ONSET OF SPECIATION

In defense of BSC, we should first emphasize that its salient feature is ‘a period of strict geographical isolation (or allopatry)’ that is needed to *complete* the process of speciation, including the evolution of RI mechanisms. Other than that, BSC does not have specific requirements either before or after the period of allopatry. In any realistic scenario of allopatric speciation, BSC would also predict ‘speciation with gene flow.’ This is because geographical events leading to full allopatry often develop gradually. For example, the closure of the Isthmus of Panama, suggested to be the strongest evidence for allopatric speciation, takes a few million years to complete [50]. Other geographical and geological events, such as the rise of a mountain range or the advance of glaciers, would also take time. In other scenarios, it may take populations thousands of years to disperse and become geographically isolated. In short, the allopatry phase may generally be preceded by a period of diminishing gene flow.

**Figure 1.**
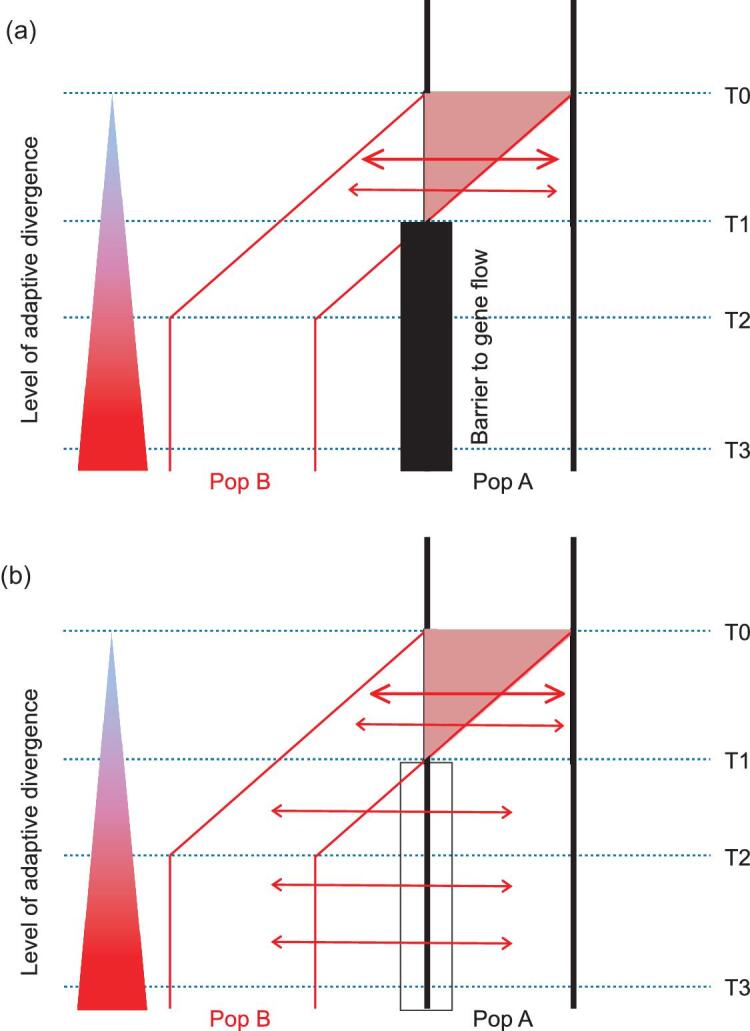
Two contrasting views of speciation. (a) The expanded model of biological species concept (BSC) that incorporates a phase of gene flow at the onset of speciation. This phase is associated with the gradual development of geographical isolation. The inference of speciation with gene flow needs to have a starting point, which is usually T0 in publications. The size of the arrow corresponds with the amount of gene flow. (b) The scenario that could reject the expanded BSC. In this scenario, gene flow continues, possibly diminished, all the way to the completion of speciation when traits of reproductive isolation have evolved.

In Fig. 1a, we depict this expanded model of BSC, which seems more realistic than generally portrayed. In this model, population B expands its range gradually between T0 and T2. Between T0 and T1, population B still overlaps with the parental population A and gene flow between the two populations only gradually diminishes. In the model conventionally portrayed, T0 – T1 would be compressed into a single time point. Such compression is neither realistic nor necessary for BSC. After T1, the two populations are truly allopatric without genetic exchanges.

 

 

Under BSC, the diverging populations may become good species at T2 by certain criteria and, by the time of T3, there is no ambiguity about their species status by most criteria. While the BSC model of Fig. 1a has a period of strict allopatry, an observer at T2 may still reach the conclusion that speciation between A and B has happened with gene flow. At T3 and beyond, the earlier signals of gene flow may dwindle to a level beyond detection. Hence, it may not be surprising that many genomic studies supporting the conjecture of speciation with gene flow are mostly about recent speciation events (see below).

## THE EVOLUTION OF RI IN RELATION TO GENE FLOW AND ALLOPATRY

Between the genomic and genic view of speciation, the main difference is on how genes interact with the environments. However, the evolution of RI, in particular, of the postmating kind, depends on the interactions among genes within the same genome. Such interactions may change the dynamics of speciation. Two contrasting views on whether gene flow during speciation can disrupt the evolution of postmating RI are presented in this section.

### Gene flow could disrupt the evolution of RI: the classical view of BSC

The BSC model portrayed in Fig. 1a leads back to the recurring question about BSC: why is geographical isolation necessary for speciation? Indeed, Darwin [1] placed much less emphasis on either geographical or RI than did Mayr [2]. By genetic reasoning, the genic view posits that speciation does not need strict allopatry as divergent selection can easily nullify the impact of gene flow [5]. (For that reason, the biological species concept should be more accurately renamed the isolation species concept.)

In Mayr's conception, geographical isolation is indispensable because the entire genome is perceived as a cohesive unit. Hence, any gene replacement by the genetic materials of a different species is bound to be deleterious. This conception may have originated in the interpretation of the genetics of postmating RI, or hybrid incompatibility (such as hybrid inviability or sterility).

The standard hypothesis is the Dobzhansky-Muller Incompatibility (DMI) model, which assumes the simplest genetic interactions possible [18,19]. In DMI, the ancestral population at the time of nascent speciation has two loci with alleles a and b, respectively. In population 1, (a, b) evolves to (A, b) and, in population 2, (a, b) evolves to (a, B). It is assumed that (A, B) causes hybrid incompatibility. By DMI, it is plausible that gene flow would impede the evolution of hybrid incompatibility. When the two populations are, respectively, accumulating the A and B alleles, any gene flow would mutually impede the spread of the new A and B alleles [18,19,34]. For example, if the immigrant (a, B) from population 2 mates with (A, b) individuals in population 1, many offspring of the new genotype (A, B) would die, making (A, b) less fit. It is true in the reciprocal direction as well. Accepting this simple argument, one may view geographical isolation to be necessary for the completion of speciation.

### Gene flow could not disrupt the evolution of RI: counter arguments from the genetics of RI

The argument above that gene flow would disrupt the evolution of postmating RI is a qualitative one. Although it is intuitively appealing, the probability of evolving postmating RI should be a function of the mutation rate, the migration/hybridization rate, the selection intensity and the nature of fitness interactions (the number of loci, the degree of gene dominance, etc.) [51].

In a simulation study [51], a model shows that postmating RI can readily evolve as long as the amount of migration does not overwhelm the selective advantage of the resident allele in its own population. The model of Yang [51] further takes advantage of the recent MIM (for migration-isolation-migration) model of speciation. He *et al*. [22] identified the speciation mechanism along the Indo-Malayan coasts to be associated with the repeated openings and closures of the Strait of Malacca. In Yang's model [51], postmating RI can evolve readily if periods of migration are interspersed with periods of isolation.

The discussion so far relies on the DMI model with two loci. This simplest model (one locus per species) uses a genetic set-up that is least conducive for the evolution of RI and most susceptible to perturbation by gene flow among possible genetic models. In a series of fine-scale dissection of hybrid male sterility in Drosophila [27–29,52–56], it is shown that hybrid incompatibility usually involves several loci per species. The two-locus DMI has not been supported by any empirical study in the literature. As explained in Fig. 8 of Cabot *et al*. [54], even a simple extension of DMI to three loci would make the evolution of postmating RI much easier.

Gavrilets [57] generalizes this view into in a ‘holey landscape,’ whereby the adaptive landscape is nearly flat with a few genotypes causing RI. These occasional genotypes represent adaptive ‘holes’ in the landscape. On this landscape, the evolution of hybrid incompatibility is relatively unimpeded as it only needs to avoid the adaptive holes. This view has extensive empirical support (see [28,29,52,55,56]; all reviewed in the last section of this perspective). Simulations by Yang [51] corroborate this view that the larger number of genes involved in RI indeed makes it easier to evolve postmating RI.

## GENETIC COMPLEXITY UNDERLYING THE EVOLUTION OF RI

The discussions above point to a curious aspect of modeling speciation – the empirical observations on the genetics of RI are often disregarded, as if they were mere details. Nevertheless, when postmating RI cannot easily evolve under a two-locus DMI model but can readily evolve under a multi-locus one, ‘god is really in the details.’ An equally instructive lesson of genetics is the modeling of premating isolation. Here, the tendency to evolve RI as a function of the number of loci is the exact opposite of evolving postmating isolation – the fewer the loci, the easier it is to evolve premating isolation. This can be demonstrated in the evolution toward the so-called ‘Fisher's equilibrium’ with only one locus.

Imagine a two-allele system with three genotypes – AA, Aa and aa. If AA and aa do not mate but all other combinations mate normally, then it is easy to see that any population would evolve in sympatry toward two isolated populations consisting solely of either AA or aa genotypes. (This is because Aa will disappear as a result of the absence of replacements from AA × aa mating.) In the theoretical models [58–61], the tendency is evident. A possible reason that premating and postmating isolation show opposite dependencies on the number of loci is the fitness reduction associated with the incompatible genotypes in the latter but not in the former.

## GENE FLOW IN RELATION TO MODES OF GEOGRAPHICAL ISOLATION

The previous sections present the genetic arguments for, as well as against, the need for a phase of isolation with no gene flow, prior to the completion of speciation (Fig. 1a). The disagreement needs to be resolved empirically. In the convention, the inference of gene flow is based on the geographical distribution of the populations. It is assumed that allopatric distribution prohibits gene flow and sympatric distribution permits unimpeded exchanges. However, such a correspondence is inexact as allopatry can be bridged by long-distance migration and sympatry may often be associated with micro-allopatry [62–66]. In either case, geography is not a good indication of gene flow, or lack thereof.

No less important, if most speciation events are parapatric between adjacent populations [67,68], it would often be ambiguous whether gene flow has happened or not during speciation. Indeed, in the MIM cycles [22], the same geographical feature is associated with episodes of gene flow and full isolation. With all these considerations, we do not use the geographical mode of isolation to assess gene flow. Instead, studies that compare genomic sequences to infer past gene flow during speciation are the main literature in this review.

## EVALUATION OF GENOMIC EVIDENCE FOR ‘SPECIATION WITH GENE FLOW’

To reject BSC of Fig. 1a, it is no longer sufficient to show that gene flow happens between T0 and the observation time, say, T2. Instead, it is necessary to show that no period of strict isolation exists between T0 and T2. Furthermore, because the duration of [T0, T1] can be variable, depending on the strength of divergent selection and the rate of gene flow, the evolutionary dynamics of this period are expected to be highly variable as well. For that reason, gene flow in the transient period of (T0, T1) can be compatible with a wide range of observations at T2, or even at T3. In Table 1, we compile publications on speciation with gene flow, aiming to identify studies that can reject BSC. In the end, we could not find compelling evidence against BSC perhaps because no study is designed to test the model of Fig. 1a. Most reports are about closely related species or subspecies, often before evolving complete RI [35–49]. In these cases, the influence of gene flow in the transient episode between T0 and T1 would be significant. We select a few examples for discussion below; other examples are listed in Table 1.

**Figure 2.**
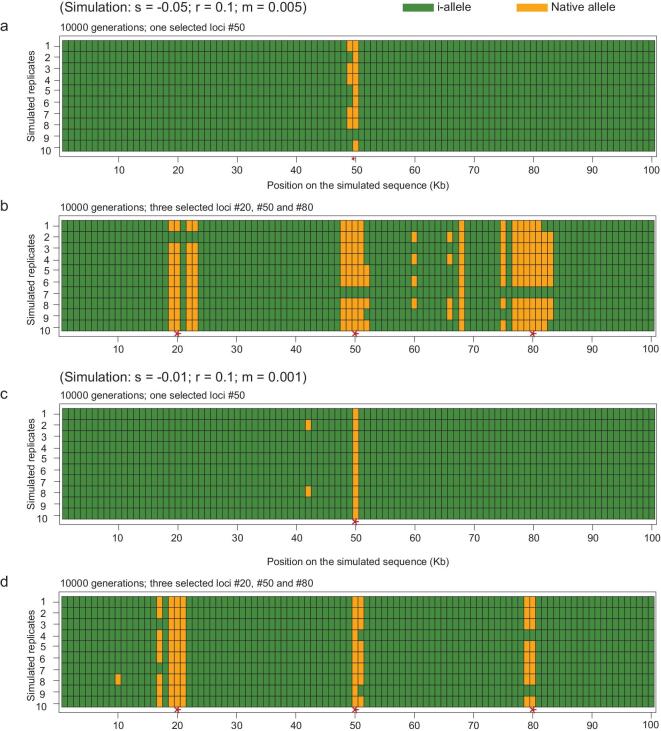
Simulated introgressions in haploid 100 kb genomes. The selected loci (or speciation genes) are marked by red stars at the bottom. Sites of introgression and non-introgression are marked green and orange, respectively. (a, b) These examples are done under strong selection (s = −0.05), low recombination (r = 0.1 for per 100 kb per generation) and high introgression (m = 0.005 per generation). (c, d) These examples are done under weak selection (s = −0.01), low recombination (r = 0.1 for per 100 kb per generation) and low introgression (m = 0.001 per generation). Note that very fine delineations of blocks are possible under the simulated conditions.

1. A common approach to population divergence is the use of the *F_ST_* statistic [35,42,46,47,49]. The *F_ST_* statistic is primarily a measure of population differentiation, rather than species divergence. For that reason, *F_ST_* is not appropriate for testing BSC. It has also been pointed out that the high *F_ST_* values in many publications are not a result of high divergence between species, but low differentiation within populations [33].
2. Many Anopholes publications report gene flow during speciation [36,43,48]. Most of them are about subspecies, or different forms of the same species. Among them, Fontaine *et al*. [36] is about a more ancient system although RI remains incomplete. It is evident that the introgressions in these species are early-stage events (see their Fig. 1C).
3. Many studies on speciation gene flow state explicitly that these are early-stage events [35,38,47]. Other studies are not informative in this respect. Nevertheless, we find no case of large-scale introgression in late stages of speciation, when postmating RI is evident. These early-stage introgressions are reported in Darwin's finches throughout their adaptive radiation [38], cichlid fish with divergent mate preferences [35] and subspecies of rabbits [47].
4. Plants often show patterns of speciation that are different from animals, in particular, in their tendencies to hybridize [37,46]. Nevertheless, we still fail to find compelling evidence from plants against the model of Fig. 1a. For example, the sunflower clades have been elegantly shown to exchange genes constantly. However, polymorphic mutations far outnumber fixed ones, suggesting the species to be in early stages of speciation (see table 2 of [46]).
5. In Martin *et al*.’s study [40] of *Heliconius*, a high incidence of genetic exchanges is reported and up to 40% of the genomes show signs of introgression across species. The authors also report long linkage disequilibrium (LD) blocks as evidence of recent introgressions. The long LD blocks indicate low density of ‘speciation genes’ in the introgression, which likely reflects nascent speciation. This topic is addressed in Fig. 2.

In a literature survey of this kind, crucial details are not always available to the readers. In cases where the authors present the diverging taxa as at an early stage, it is possible to stage them close to T1-T2. In cases where the taxa are close to T3 (e.g. when strong postmating RI has evolved), it is more difficult to see whether the model of Fig. 1a or b applies. In the next section, we attempt to make a distinction between the T3 stages of the two models.

## CAN BSC BE REJECTED? STUDIES OF GOOD SPECIES WITH STRONG RI

The reviews of the literature above lead to the central question – what might constitute the necessary evidence against the BSC model of Fig. 1a, if BSC is indeed a faulty model?

A starting point should be the use of good species that are delineated by strong RI mechanisms and, hence, no longer in the nascent stage of speciation. In this section, we review the detailed genetic analysis of RI in the *Drosophila* literature, whereby functional tests of speciation genes have been done [28,29,52–56]. These studies show that, between sibling species that are morphologically indistinguishable, the number of ‘speciation loci’ is already high, in the hundreds or thousands [53–56]. Such a high density of adaptive differences would yield a distinct introgression pattern if gene flow continues toward the very end of speciation, as shown by the simulations in Fig. 2.

### The number of speciation loci: from behavioral races to good species of *Drosophila*

This question can only be answered by detailed genetic analyses from incipient species all the way to ‘good species.’ Phenotypic observations suggest that even closely related species differ by a multitude of traits. When such traits were dissected at the genetic and molecular levels, each was found to be highly polygenic [42,49,69–73], implying extensive genetic divergence. A particularly instructive case is hybrid sterility in *Drosophila*. Among sibling species that show little morphological divergence, the number of genes involved in hybrid male sterility, that is spermatogenic failure, is in the hundreds [27,29,53–56]. For example, in the identification of hybrid sterility loci between *D. melanogaster* and *D. simulans*, Sawamura *et al*. [53] used small chromosomal deficiencies to uncover the sterility loci. To their surprise, every single deficiency uncovered at least one such locus. Extrapolating to the whole genome, they estimate thousands of speciation genes between these two best-known sibling species.

The cloning of the component genes further reveals a complex web of interactions [28,52]. This level of divergence means that selection, presumably sexual in nature, drives spermatogenic programs to evolve extensively and rapidly [74] with hybrid sterility being the incidental byproduct. Other ‘speciation traits’ such as sexual isolation, genital morphology and neural development depict a genetic basis that is qualitatively similar [75–77]. Again, a detailed account of mating loci between the African and cosmopolitan behavioral races of *D. melanogaster* shows that the genetic differentiation in premating isolation may be even more extensive than hybrid male sterility [75,78]. The number of loci responsible for the differentiation in the genital morphology between *D. simulans* and *D. mauritiana* (closer to *D. simulans* than *D. melanogaster*) is estimated to be >15 [76]. While the data are from the genus of *Drosophila*, which offer the rarely attained resolution, the genetic basis of hybrid sterility does follow rules applicable to a wide range of taxa [9] from vertebrates (birds and mammals) to invertebrates (*Drosophila* and butterflies). If we extend the definition of speciation loci to genes that cause fitness reduction upon introgression, a definition most appropriate for introgression analysis, the number of speciation loci becomes even larger. Fang *et al*. [30] showed that introgressions without any detectable phenotypes may often harbor strong fitness-reducing genes when the fitness is measured in long-term population experiments.

In conclusion, the extent of functional divergence at the genic level is very high, even among sibling species that look identical. The substantial genic divergence between good species may hold the key to the expected pattern of introgression in late stages of speciation.

### Expected genomic patterns of introgressions when speciation is close to completion

The literature on gene flow during speciation is, by and large, compatible with Fig. 1a, and, hence, is also compatible with BSC. In contrast, the scenario of Fig. 1b, whereby gene flow continues until the completion of speciation, is incompatible with the core of BSC.

What might be the resultant genomic patterns, if gene flow continues as depicted in Fig. 1b? We carry out computer simulations with unceasing gene flow. The model clearly violates the core assumptions of BSC. In the simulation of a stretch of DNA 100 kb long, the populations are assumed to have diverged with one ‘speciation locus’ at T2 and three speciation loci at T3. For simplicity, gene flow occurs in only one direction. For introgression to be feasible, either the migration rate (m) has to be sufficiently high (m = 0.005, Fig. 2a and b) and/or selection against the speciation allele has to be weak (s = 0.01; Fig. 2c and d). With the genetic input from migration, which is subjected to removal by natural selection, the genetic makeup would eventually reach a steady state. The steady-state results are presented in Fig. 2. As expected (but shown only in Supplementary Fig. S1), when migration is low (m = 0.001) and selection is strong (s = −0.05), stable introgression is not observed.

Figure 2 shows two large introgressions at T2 but, at T3, these two introgressions are broken into at least four much smaller segments. In other words, when gene flow and selection reach an equilibrium, the introgressions become much more fine-grained, effectively reflecting the number of speciation loci accrued. Such fine-grained introgressions have not been reported in the literature as most experimental designs rely on the statistical power inherent in large segments of DNA. Fine-grained introgressions are therefore not possible to detect in most studies. Furthermore, the fine-grained pattern represents a balance between gene flow and natural selection, but such a balance is also transient (and difficult to find) in nature. In a companion paper, Wang *et al*. [79] were able to find such a speciation event, thanks to several factors including the geographical distribution of populations and the duration of gene flow. The confluence of so many factors, necessary for testing BSC rigorously, may not be common.

## THE IMPORTANCE OF A DEFINITIVE ACCEPTANCE, OR REJECTION, OF BSC

A realistic BSC model would require a period of strict isolation for speciation to complete its course. As shown in Fig. 1a, this BSC model must also incorporate a transient episode for genetic exchanges while geographical isolation is developing. Therefore, the proper test of BSC should be about how speciation ends (after a long period of allopatry with no gene flow), rather than how speciation begins (most likely with gene flow that diminishes in time). The extensive literature documenting gene flow during speciation is largely about speciation starting with a phase of gene flow [35–49]. In this sense, the collective evidence is akin to the ‘exceptions proving the rule (of BSC).’ The difficulty in rejecting BSC does not necessarily mean that strict allopatry is indeed the main mechanism of speciation. Genetic reasoning suggests that RI or ecological differentiation may easily evolve under continuous gene flow [5–7]. In Fig. 2, a possible test is proposed.

It would be wrong to perceive BSC (and its definitive acceptance or rejection) as no more than an issue of historical interest. The issue is fundamental because it is about the genetic nature of species. BSC is conceived on the assumption of strong cohesiveness of the entire genome. By BSC, any genetic exchange between species should be maladaptive [2,5]. Modern genetics with all the transgenic experiments between species may seem to reject BSC as overly rigid as the transgenes usually function well in another species (unless the genes were chosen specifically for demonstrating interspecific differences; e.g. [28,52,77]). However, whether most randomly chosen genes have no fitness effects across species is unknown because the fitness decline only needs to be stronger than the strength of genetic drift, which is often $\ll$ 10^−3^. As demonstrated by Fang *et al*. [30], the absence of phenotypic effects associated with interspecific exchanges can have a fitness consequence measurable in long-term laboratory populations. If such observations are common, most genomes probably evolve with the broad cohesiveness beyond the resolution of modern molecular experiments that focus on large and measurable differences.

The resolution of the debate on BSC is important on many fronts of evolutionary biology. For example, in the taxonomic practice of naming species, BSC has not been highly relevant. This disconnect between adjacent fields has been a central dilemma in evolutionary biology [3–5,80,81]: do we or do we not accept the naming of new species that are not based on the (biological) species concept? Similarly, in the study of biodiversity, speciation mechanisms have not been relevant either. For example, a global hotspot of coastal biodiversity is in the Indo-western Pacific [82]. As pointed out by He *et al*. [22], the rejection that speciation mechanisms do not play a role in driving this spectacular biodiversity is the absence of geological features in the region that can impose the required geographical isolation. Ironically, the best-known feature of geographical isolation (i.e. the Isthmus of Panama) does not drive unusual biodiversity between the Pacific and Atlantic coasts. The disconnect between biodiversity and speciation analyses appears to be rooted in the conception of BSC on how species are formed.

There are many other discrepancies that reflect the dominance of BSC in modern evolutionary biology. The discrepancies can be resolved only when BSC is convincingly accepted or rejected. As stated, cases of sympatric speciation (e.g. [62–66]) would be a powerful rejection of BSC if micro-allopatry could be ruled out and gene flow could be demonstrated. Finally, the MIM cycle model cited above [22] offers a novel alternative, whereby phases of isolation and migration are interspersed during speciation. MIM cycles, a rejection of BSC, nevertheless retain some of its key features [22]. Many newer approaches to testing BSC will be needed, as a recent example shows [79].

## Supplementary Material

nwz220_Supplemental_File
