# Supplementary material for: Genes and speciation: is it time to abandon the biological species concept?
Source: Natl Sci Rev. 2019 Dec 31;7(8):1387–97. doi: 10.1093/nsr/nwz220 (PMC8288927; doi:10.1093/nsr/nwz220)
Supplement: nwz220_Supplemental_File [file nwz220_supplemental_file.docx]

**Supplementary figure:**


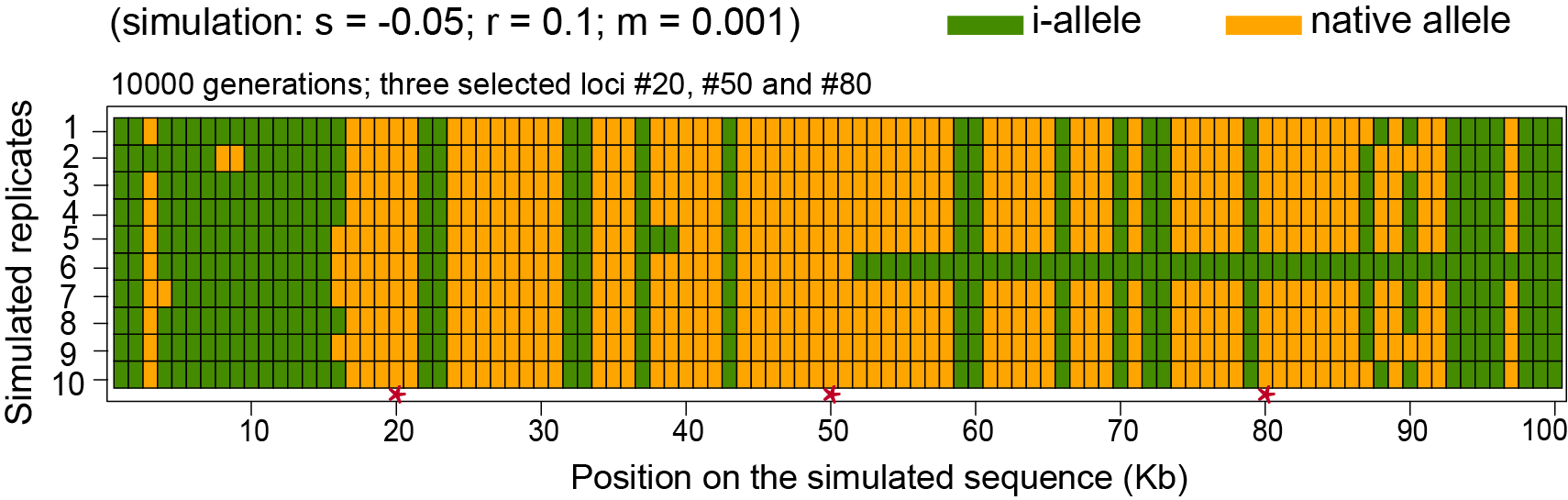


**Figure S1.** Simulated introgressions in haploid 100 Kb genomes. This example is done under strong selection (s = -0.05), low recombination (r = 0.1 for per 100Kb per generation) and low introgression (m=0.005 per generation). Note that stable introgression is not observed. The selected loci (or speciation genes) are marked by red stars at the bottom. Sites of introgression and non-introgression are marked green and orange, respectively.
